# Phylogenetic diversity and antioxidant activities of culturable fungal endophytes associated with the mangrove species *Rhizophora stylosa* and *R*. *mucronata* in the South China Sea

**DOI:** 10.1371/journal.pone.0197359

**Published:** 2018-06-13

**Authors:** Jing Zhou, Xiaoping Diao, Tao Wang, Guangying Chen, Qiang Lin, Xiaobo Yang, Jing Xu

**Affiliations:** 1 Institute of Tropical Agriculture and Forestry, College of Material and Chemical Engineering, Hainan University, Haikou, P. R. China; 2 Key Laboratory of Tropical Medicinal Plant Chemistry of Ministry of Education, Hainan Normal University, Haikou, P. R. China; Tallinn University of Technology, ESTONIA

## Abstract

Mangrove endophytic fungi can produce impressive quantities of metabolites with promising antioxidant activities that may be useful to humans as novel physiological agents. In this study, we investigated the phylogenetic diversity and antioxidant potential of 46 fungal endophytes derived from the mangrove species *Rhizophora stylosa* and *R*. *mucronata* from the South China Sea. The fungal isolates were identified using a combination of morphological characteristics and phylogenetic analysis of the internal transcribed spacer (ITS) sequences. Seventeen genera belonging to 8 taxonomic orders of Ascomycota were discovered, specifically, *Botryosphaeriales*, *Capnodiales*, *Diaporthales*, *Eurotiales*, *Glomerellales*, *Hypocreales*, *Pleosporales*, and *Xylariales*. The most abundant fungal orders included Xylariales (35.49%) and Diaporthales (27.61%), which were predominantly represented by the culturable species *Pestalotiopsis* sp. (34.54%) and *Diaporthe* sp. (18.62%). The stems showed more frequent colonization and species diversity than the roots, leaves, hypocotyls, and flower tissues of the host plant. The antioxidant activities of all the isolated fungal extracts on four different culture media were assessed using improved 2,2′-diphenyl-1-picrylhydrazyl (DPPH) and 2,2′-azino-bis(3-ethylbenzothiazoline-6-sulphonicacid) (ABTS) methods. A relatively high proportion (84.8%) of the isolates displayed antioxidant capacity (%RSA > 50%). Further research also provided the first evidence that HQD-6 could produce flufuran as a significant radical scavenger with IC_50_ values of 34.85±1.56 and 9.75±0.58 μg/mL, respectively. Our findings suggest that the utilization of a biotope such as that of the endophytic fungal community thriving on the mangrove plants *R*. *stylosa* and *R*. *mucronata* may be suitable for use as a sustainable resource for natural antioxidants.

## Introduction

Severe oxidative damage from free radicals is associated with various conditions including cancer, inflammation, aging, and neurodegenerative diseases. Elucidating the vital role that antioxidants play in the prevention of reactive-oxygen-species-mediated tissue damage and in the enhancement of human health is thus warranted [1, 2]. Dietary antioxidants are believed to contribute specific effects that prevent specific health risks associated with oxidative-stress-related diseases and undesired transformations [3]. Currently, some artificially synthesized antioxidants such as butylated hydroxytoluene (BHT), butylated hydroxyanisole (BHA), and tert-butylhydroquinone (TBHQ) have been used to scavenge radicals in foods and biosystems. However, the use of these chemical substances has become limited over time due to their risks to human health, which include protein and DNA damage, toxicity, and other effects [4, 5]. Consequently, there has been a growing interest in natural antioxidants from microbes as a sustainable resource that may contribute towards the attenuation of oxidative damage.

Fungal endophytes, which have been found to be ubiquitously distributed in nearly 300,000 plant species, complete at least part of their life cycle within healthy plant tissues without causing any apparent disease [6–8]. Most previous studies on endophytic fungi have focused on land plants, whereas recent investigations have focused on the discovery of unique and diverse endophytic fungi living on organisms that inhabit specialized and poorly characterized biotopes, such as mangrove habitats [9–11]. Mangrove endophytic fungi, which comprise the second largest ecological group of marine fungi, promote the survival of host plants under harsh environmental conditions through long-term plant-fungi interactions [12, 13]. For example, they may confer tolerance to various stresses such as salinity, oxidative stress, hyperosmotic stress, and drought and nutrient stress [14, 15]. Ever since Cribb first described endophytic fungi isolated from mangrove roots in 1955, several studies have been conducted on the fungi living in mangroves along the coastlines of the Indian, Pacific, and Atlantic Oceans have been conducted [16]. Hyde listed approximately 120 fungal species that colonize 29 mangrove plants globally, including 87 ascomycetes, 31 mitosporic fungi, and two basidiomycetes [17]. Schmit and Shearer reported 625 mangrove-associated fungi including 279 ascomycetes, 277 mitosporic fungi, 29 basidomycetes, three chytridomycetes, two myxomycetes, 14 oomycetes, nine thraustochytrids, and 12 zygomycetes [18, 19]. According to the frequency with which they appear, *Alternaria*, *Aspergillus*, *Cladosporium*, *Clolletotrichum*, *Fusarium*, *Paecilamyces*, *Penicillium*, *Pestalotiopsis*, *Phoma*, *Phomopsis*, *Phyllosticta*, and *Trichodema* have been recognized as the predominant culturable endophytic fungi in mangroves [20].

Endophytic fungi from mangroves are a relatively underappreciated reservoir of bioresources. They have been evaluated as potential pharmaceutical and agricultural resources. Recent studies have investigated the biodiversity and distribution of mangrove endophytic fungi in the South China Sea. The taxonomic identities and diversity of endophytic fungal communities isolated from five species of the genus *Sonneratia*, namely, *S*. *caseolaris*, *S*. *hainanensis*, *S*. *ovata*, *S*. *Paracaseolaris*, and *S*. *apetala*, and four species of Rhizophoraceae, namely *Ceriops tagal*, *R*. *apiculata*, *R*. *stylosa*, and *Bruguiera sexangula* var. *rhynchopetala* have been addressed [21]. Although endophytic fungi from the abovementioned mangrove species have already been found on leaves, the fungal diversity and associated antioxidant potential of the various tissues of *R*. *stylosa* and *R*. *mucronata* in the South China Sea have only rarely been studied.

In an attempt to identify antioxidants from natural resources [22, 23], we decided to investigate the antioxidant potential of endophytic fungi derived from the mangrove species *R*. *stylosa* and *R*. *mucronata* (Rhizophoraceae) in the South China Sea. Fungal diversity was evaluated using a combination of morphologic characteristics and phylogenetic analysis of internal transcribed spacer (ITS) sequences. Antioxidant radical scavenging capacity was measured by using 2,2′-diphenyl-1-picrylhydrazyl (DPPH) and 2,2′-azino-bis(3-ethylbenzothiazoline-6-sulphonicacid) (ABTS) assays. This study aims to provide complete information on the *in vitro* antioxidant potential of the endophytic fungi of these two hosts.

## Materials and methods

### Sampling site and plant material

Healthy roots, stems, leaves, hypocotyls and flowers of *R*. *stylosa* (Rhizophoraceae) and roots, stems, leaves and flowers of *R*. *mucronata* were collected from a specific location (110°32′-110°37′ E, 19°51′-20°01′ N) in Dong Zhai Gang-Mangrove Garden on Hainan Island, China. The collected plants were authenticated by Prof. Dr. Xiaobo Yang (Hainan University), with a voucher specimen preserved in the herbarium of College of Landscape and Horticulture, Hainan University.

### Isolation of endophytic fungi

To obtain the endophyte within different parts of the plant, surface sterilization was carried out following an isolation protocol described by Proksch’s group [24] with some modifications. Samples were initially washed with tap water, then surface sterilized by dipping in 75% ethanol for 1 min, in bleach solution containing 1.3% sodium hypochlorite for 3 min, in 75% ethanol for 30 s and finally were rinsed three times (3 min each) in sterile Millipore water. The surface sterilized samples were cut into small segments using a sterile scalpel and were incubated on PDA plates supplemented with streptomycin to suppress bacterial growth at 28°C in darkness for 2–4 weeks and were checked daily for hyphal growth. Single isolate were transferred to fresh PDA plates using the hyphal tip method. Culture purity was determined from colony morphology. Voucher strains are deposited at one of the authors’ laboratory (J. X.). http://dx.doi.org/10.17504/protocols.io.jhmcj46 [PROTOCOL DOI]

### Identification of endophytic fungi

The endophytic fungi were identified in combination of morphologic characteristics and p internal transcribed spacer (ITS) sequences.

### DNA extraction, PCR amplification, sequencing and phylogenetic analysis

Genomic DNA was extracted from fresh fungal mycelium collected in a PDA plate using cetyltrimethylammonium bromide (CTAB) method [25]. The internal transcribed spacer (ITS1-5.8S-ITS2) regions of the fungi were amplified with the universal ITS primers, ITS1F (5′ CTTGGTCATTTAGAGGAAGTAA 3′) and ITS4 (5′ TCCTCCGCTTATTGATATGC 3′) [26], using the polymerase chain reaction (PCR). The amplified products were submitted for sequencing (Invitrogen, Shanghai) and aligned with the sequences in the GenBank by Basic Local Alignment Search Tool (BLAST) programs to find out the sequence homology with closely related organisms. When the top three matching BLAST hits were from the same species and were ≥ 98% similar to the query sequence, this species name was assigned to the selected isolate. Fungal ITS-rDNA sequences of 46 representative isolates were deposited in GenBank under the accession number KX618209 and numbers ranging from KX631698 to KX631742 are provided in Table 1. Phylogenetic analyses of the endophytes were carried out using MEGA 5.1 software. The neighbor-joining (NJ) method was used to infer the evolutionary history of the fungal isolates, and the bootstrapping was carried out using 1,000 replications [27]. http://dx.doi.org/10.17504/protocols.io.jinckde [PROTOCOL DOI]

**Table 1:** Composition of media used for stationary fermentation of mangrove associated culturable fungi.

PDA	200g potato	CZA	30g sucrose
	20g glucose		15g agar
	1000ml seawater		0.5g KCl
	PH = 7.0		1g K_2_HPO_4_
			1.5g MgSO_4_·7H_2_O
			0.01g FeSO_4_
			1000ml seawater
			pH = 7.5
RM	100g rice	GM	7.5g grain
	0.6g peptone		7.5g bran
	0.1g KH_2_PO_4_		0.5g yeast extract
	0.1g CaCl_2_,		0.1g sodium tartrate
	0.5g MgSO_4_		0.01 FeSO_4_·7H_2_O
	100ml H_2_O		0.1g sodium glutamate
			0.1ml pure corn oil
			30ml H_2_O

### Fermentation and extraction

The fungal strains were cultured on Petri dishes of potato dextrose agar (PDA) at 28°C for 5 days. The agar patches with the title fungus were inoculated into Erlenmeyer flasks (1000 mL). To monitor the influences of the fermentation media towards the fungal production of antioxidant metabolites, four different media (Table 1) such as Potato Dextrose Agar (PDA), Czapek’s Agar (CZA), Rice Medium (RM), Grain Medium (GM) were adopted for the stationary fermentation and grown for 40 days at room temperature. The harvested cultures were extracted twice with an equal volume of EtOAc and evaporated to dryness and residues were dissolved in dimethyl sulfoxide (DMSO) to give a stock solution (10 mg/mL) for antioxidant assays. http://dx.doi.org/10.17504/protocols.io.jipckdn [PROTOCOL DOI]

### Isolation of flufuran

HQD-6 was fermentated on rice solid medium (to 100 g commercially available rice was added 110 mL of distilled water and kept overnight prior to autoclaving, 100 flasks) at room temperature under static conditions and daylight for 40 days. The mycelia and solid rice medium were extracted with EtOAc (each 200 mL) and the combined EtOAc extracts were evaporated under reduced pressure to yield 62.0 g residue. This residue was separated into seven fractions (Fr. 1- Fr. 7) on a vacuum liquid chromatography (VLC) on a short silica gel column using a step gradient elution of CH_2_Cl_2_/MeOH (v/v 0:100–100:0). Fr. 3 (5.0 g) was rechromatographed on a silica gel column, eluted with petroleum petroleum ether/CH_2_Cl_2_/MeOH (5:5:0.1), to provide four subfractions (fractions 3.1–3.4). Promising Fr. 3.3 (126 mg) was processed to further chromatographic separation using Sephadex LH-20 with CHCl_3_/MeOH (5:5:0.1) as eluent to afford flufuran (10.0 mg). http://dx.doi.org/10.17504/protocols.io.n9zdh76 [PROTOCOL DOI]

### Assay for antioxidant activity

#### DPPH radical scavenging capacity measurement

The radical scavenging ability was estimated by using adapted 2,2'-diphenyl-b-picrylhydrazyl (DPPH) method described previously [22, 28]. Thus, an aliquot of extract (10 μl) was added to 195 μl of ethanolic DPPH (120 μM). The reaction mixtures were pipetted on 96-well microtitre plates and incubated at room temperature for 30 min in the dark and absorbance was measured at 517 nm. http://dx.doi.org/10.17504/protocols.io.jiqckdw [PROTOCOL DOI]

#### ABTS radical scavenging capacity measurement

Free radical scavenging capacity using a stable ABTS radical was performed according to a modification of the improved ABTS method of Tian & Schaich [29]. The assay was carried out in a 96-well microtitre plates. Extract (10 μl) or ethanol (10 μl, control) was added to 195 μl ABTS radical solution and allowed to react for 30 min until a stable absorbance was obtained. The decrease in absorbance at 734 nm was measured against a blank (ethanol). http:// dx.doi.org/10.17504/protocols.io.jirckd6 [PROTOCOL DOI]

### Statistical analysis

The relative species frequency (RF) was used to estimate a specific endophytic taxon from mangrove tissue or individual plant and was calculated as the number of isolates of an individual species from the assemblage divided by the total number of isolates of all species [30]. Camargo’s index (1/*S*) was used to determine fungal dominance, A species is defined as dominant when satisfy *RF*> 1/*S*, where *S* represents species richness, equals the total number of species in an assemblage [31]. The colonization frequency (CF%) can reflect the extent of endophyte infection was calculated as *CF* = (*N_col_*/*N_t_*)×100, where *N*_*col*_ is the number of segments colonized by an individual species, and *N*_*t*_ is the total number of incubated segments[32].

The fungal endophytic diversity of each mangrove variety and the different plant tissues was estimated with Shannon–Weine Diversity index(*H*′), Simpson’s Diversity index(1-*D*), Shannon eveness (*E*) and Sorensen’s index of similarity (*QS*) using the software EstimateS version 9.10, nonparametric indices abundance-based coverage estimator (ACE), Chao1 and Chao2 were adopted[33].

Results were expressed as mean ± standard deviation (SD) of triplicate of measurements for the DPPH and ABTS assays. Data were analyzed by ANOVA (*p* < 0.05) using Statistical Analysis System (SAS, version 9.1).

## Results and discussion

### Phylogenetic diversity of culturable fungi derived from *R*. *stylosa*

A total of 135 isolates were recovered from 375 fragments of *R*. *stylosa*. Based on morphological characteristics, 25 independent representatives were selected for sequencing (S1 Fig). These isolates were identified based on ITS sequences or their closest neighbors retrieved from NCBI. BLAST analysis indicated that the majority of the isolated endophytes displayed diverse taxonomic affinities. Phylogenetic reconstruction based on the neighbor-joining (NJ) algorithm indicated that they could be classified into five taxonomic orders: Botryosphaeriales, Capnodiales, Diaporthales, Hypocreales, and Xylariales. Some representatives of the predominant order, Xylariales, including isolates from the genera *Pestalotiopsis* and *Seiridium*, were recovered. This is because they rapidly sporulate and are easily cultured. Diaporthales species were mainly isolated from stem tissues and represented by the genera *Phomopsis* sp., *Diaporthe*, *Valsa*, and *Cytospora*. Botryosphaeriales, represented by the genera *Botryosphaeria*, *Lasiodiplodia*, *Neofusicoccum*, and *Phyllosticta*, grew widely across the different tissues. The least abundant orders were Capnodiales and Hypocreales, which were represented by the genera *Cladosporium* and *Fusarium*, respectively. Among the 25 fungal isolates identified, the predominant fungi observed were *Neopestalotiopsis protearum* (31.11%), *Pestalotiopsis* spp. (11.84%), *Phomopsis longicolla* (8.15%), *Diaporthe perseae* (7.41%), *Neofusicoccum mangiferae* (6.67%), *Diaporthe* sp. (5.19%), and *Lasiodiplodia pseudotheobromae* (5.19%) (Table 2).

**Table 2:** Phylogenetic affiliations of cultivable fungi associated with mangroves *R*. *stylosa* and *R*. *mucronate*.

Isolate ID^a^	Order	Genus	Closest identified relative	Identity (%)	Accession number	Ovelap (bp)	RF(%)					
							Root	Stem	Leaf	Hypocotyl	Flower	Total
HHL31	Botryosphaeriales	*Lasiodiplodia*	*Lasiodiplodia pseudotheobromae*(GQ469969)	99	KX631698	517	-	5.19	-	-	-	5.19
HHL94			*Lasiodiplodia theobromae*(KM406107)	99	KX631699	531	-	0.74	-	0.74	-	1.48
HHL96		*Phyllosticta*	*Guignardia mangiferae*(JQ341114)	99	KX631700	634	-	-	0.74	-	-	0.74
HHL70		*Botryosphaeria*	*Botryosphaeria dothidea*(KC492490)	99	KX631701	540	1.48	-	-	0.74	-	2.22
HHL75		*Neofusicoccum*	*Neofusicoccum parvum*(LN832409)	99	KX631702	548	1.48	-	-	-	-	1.48
HHL129			*Neofusicoccum mangiferae*(KF479466)	99	KX631703	557	5.19	-	-	0.74	0.74	6.67
HHL104	Capnodiales	*Cladosporium*	*Cladosporium cladosporioides*(KT336505)	100	KX631704	508	-	-	0.74	0.74	-	1.48
HHL55	Diaporthales	*Cytospora*	*Cytospora rhizophorae*(DQ996040)	99	KX631705	577	-	1.48	-	-	-	1.48
HHL59		*Diaporthe*	*Diaporthe ceratozamiae*(JQ044420)	99	KX631706	533	-	0.74	-	-	-	0.74
HHL53			*Diaporthe eucalyptorum*(KR183772)	97	KX631707	545	-	2.96	-	-	-	2.96
HHL61			*Diaporthe perseae*(KC343173)	99	KX631708	553	-	2.96	-	4.44	-	7.41
HHL7			*Diaporthe sp*.(EU330620)	99	KX631709	544	-	1.48	-	2.96	0.74	5.19
HHL22		*Phomopsis*	*Phomopsis asparagi*(KF498860)	99	KX631710	533	-	-	-	0.74	0.74	1.48
HHL52			*Phomopsis glabrae*(AY601918)	96	KX631711	551	-	0.74	-	-	-	0.74
HHL50			*Phomopsis longicolla*(EU236702)	99	KX631712	535	1.48	5.93	-	-	0.74	8.15
HHL20			*Phomopsis sp*.(EF488377)	99	KX631713	554	0.74	-	-	0.74	0.74	0.74
HHL81		*Valsa*	*Valsa brevispora*(FJ487920)	97	KX631714	574	-	0.74	-	-	-	0.74
HHL48	Hypocreales	*Fusarium*	*Fusarium solani*(KJ174390)	99	KX631715	540	0.74	-	-	-	-	0.74
HHL56	Xylariales	*Pestalotiopsis*	*Pestalotiopsis theae*(JX436804)	99	KX631716	531	-	0.74	-	-	-	0.74
HHL46			*Neopestalotiopsis protearum*(KR183783)	99	KX631717	514	3.70	8.89	0.74	17.78	-	31.11
HHL82			*Pestalotiopsis microspora*(AF377296)	99	KX631718	574	-	0.74	-	-	-	0.74
HHL51			*Pestalotiopsis palmarum*(AF409990)	100	KX631719	509	0.74	-	-	-	-	0.74
HHL79			*Pestalotiopsis photiniae*(GU395992)	100	KX631720	524	-	1.48	-	1.48	-	2.96
HHL10			*Pestalotiopsis sp*.(EF451799)	100	KX631721	524	2.96	4.44	-	4.44	-	11.84
HHL38		*Seiridium*	*Seiridium ceratosporum*(AY687314)	98	KX631722	561	-	0.74	-	-	-	0.74
HQD83	Botryosphaeriales	*Botryosphaeria*	*Botryosphaeria fusispora*(JX646789)	100	KX631723	575	-	3.49	-	1.16	-65	4.44
HQD72		*Lasiodiplodia*	*Lasiodiplodia theobromae*(KR183781)	99	KX631724	485	10.47	5.81	-	1.16	17.44	16.67
HQD23		*Neofusicoccum*	*Neofusicoccum mangiferae*(KF479465)	100	KX631725	536	-	5.81	4.65	4.65	-5.11	14.44
HQD41			*Neofusicoccum parvum*(FJ904817)	99	KX631726	539	-	4.65	-	1.16	-81	5.56
HQD47		*Pseudofusicoccum*	*Pseudofusicoccum stromaticum*(FJ441621)	99	KX631727	546	1.16	-	-	-	-6	1.11
HQD55	Pleosporales	*Paracamarosporium*	*Paraconiothyrium hawaiiense*(KF498872)	99	KX631728	538	-	1.16	-	-	-	1.11
HQD24	Eurotiales	*Aspergillus*	*Aspergillus fumigatus*(KP724998)	100	KX618209	547	-	-	-	-	1.16	1.11
HQD48	Hypocreales	*Fusarium*	*Fusarium verticillioides*(KJ957786)	100	KX631729	515	-	-	-	-	1.16	1.11
HQD62	Diaporthales	*Diaporthe*	*Diaporthe eucalyptorum*(KR183772)	97	KX631730	546	1.16	1.16	-	-	-	2.22
HQD33			*Diaporthe pascoei*(JX862532)	99	KX631731	546	4.65	5.81	-	-	-	10.47
HQD17			*Diaporthe phaseolorum*(AF001017)	99	KX631732	544	1.16	-	-	-	-	1.11
HQD29			*Diaporthe sp*.(KJ490597)	99	KX631733	540	4.65	2.33	-	-	-	11.11
HQD57		*Phomopsis*	*Phomopsis glabrae*(AY601918)	99	KX631734	529	1.16	1.16	-	-	-	2.22
HQD8			*Phomopsis longicolla*(EU236702)	100	KX631735	534	-	1.16	-	-	-	1.11
HQD22		*Valsa*	*Valsa brevispora*(FJ487920)	100	KX631736	570	-	-	-	-	1.16	1.11
HQD25	Glomerellales	*Colletotrichum*	*Colletotrichum gloeosporioides*(KP145437)	99	KX631737	536	4.65	-	-	-	-	4.44
HQD28	Xylariales	*Eutypella*	*Eutypella scoparia*(JF894102)	99	KX631738	568	1.16	-	-	-	-	1.11
HQD5		*Pestalotiopsis*	*Neopestalotiopsis protearum*(KR183783)	99	KX631739	512	-	8.14	-	-	-	7.78
HQD20			*Pestalotiopsis microspora*(AF377296)	99	KX631740	567	-	1.16	-	-	1.16	2.22
HQD1			*Pestalotiopsis protearum*(JX556231)	100	KX631741	514	-	2.33	-	-	-	2.22
HQD6			*Pestalotiopsis sp*.(FJ487914)	100	KX631742	559	-	6.98	-	-	1.16	7.78

^a^ Isolates with prefix HHL and HQD are isolated from *R*. *stylosa* and *R*. *mucronata*, respectively.

**Fig 1:**
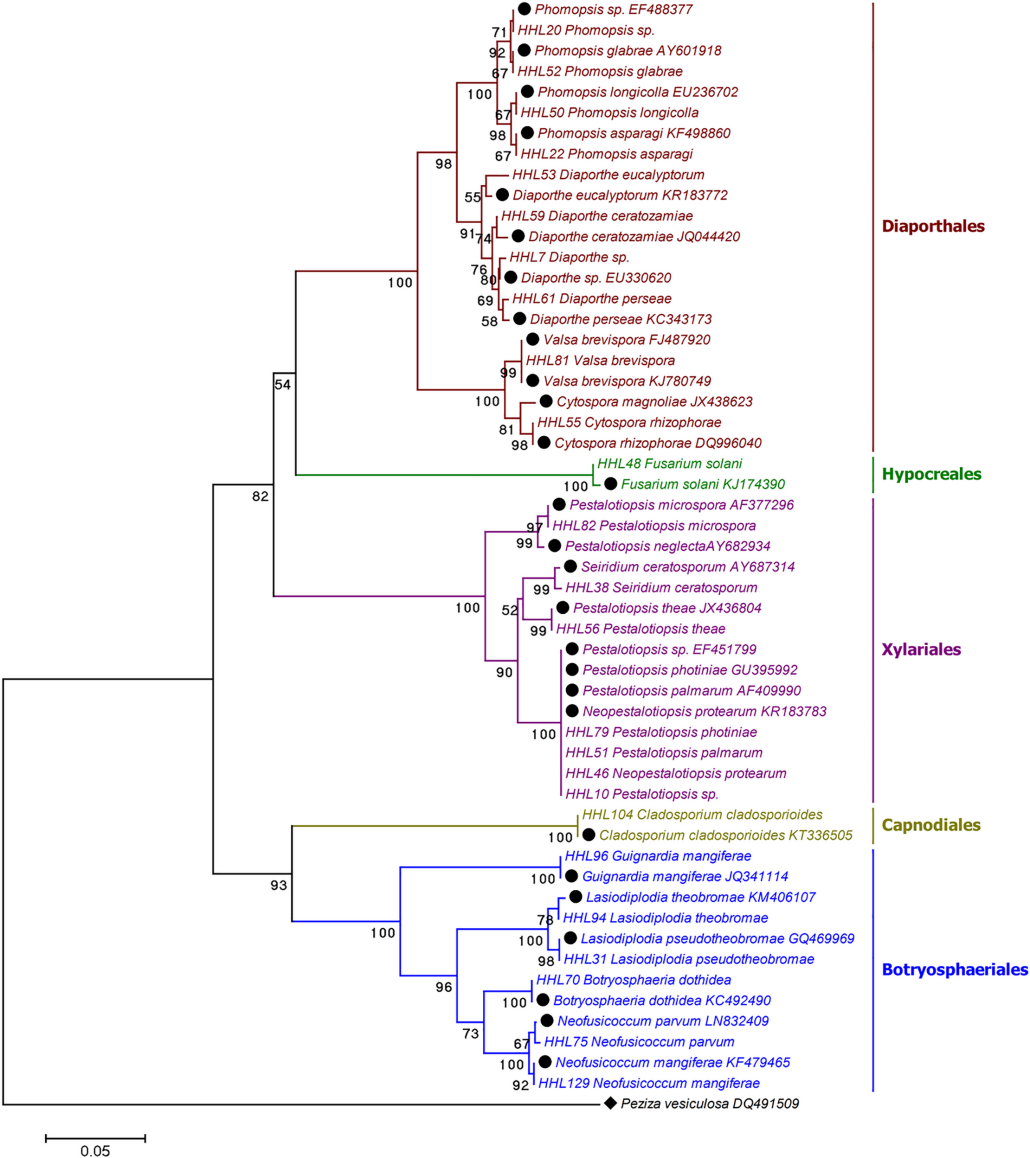
Neighbor-joining phylogenetic tree of 25 representative isolates from *R*. *stylosa* based on ITS gene sequences. The values at each node represent the bootstrap values from 1,000 replicates, and the scale bar represents 0.05 substitutions per nucleotide. *Peziza vesiculosa* DQ491509 served as an outgroup.

**Fig 2:**
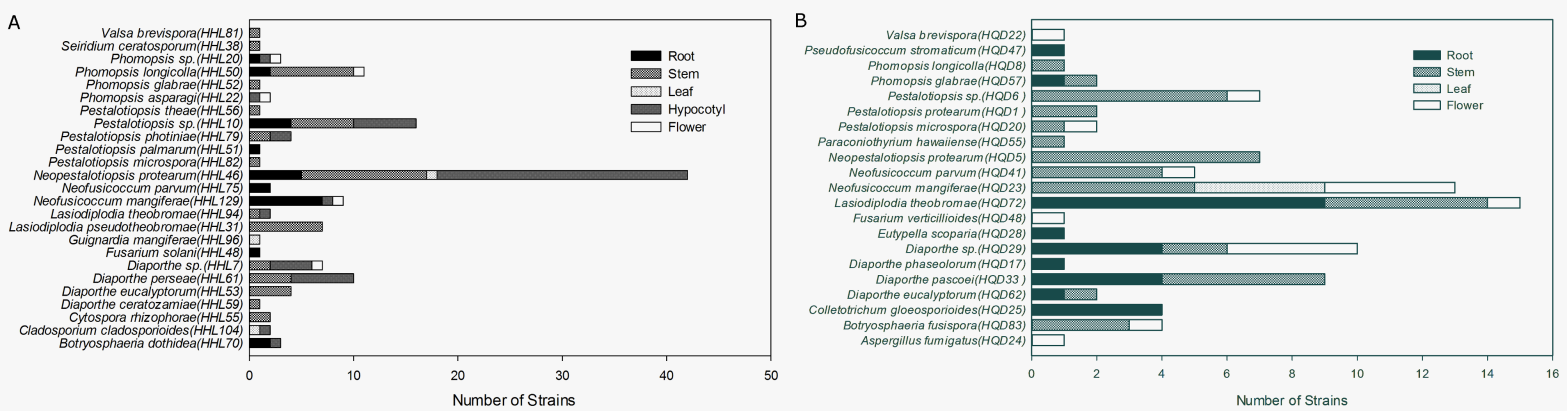
Distribution of fungal genera present in different tissues of (A) *R*. *stylosa* and (B) *R*. *mucronata*. Each value is expressed as mean ± standard deviation (*n* = 3).

The highest endophytic fungi colonization frequency was observed in the stems (CF = 53.33%), followed by the hypocotyls (49.33%) and roots (25.33%). The lowest frequencies were observed in the flowers (6.67%) and leaves (4.00%) (Table 3). As assessed using Camargo’s index, the predominant endophytic fungal species differed between each tissue, with the exception of *Neopestalotiopsis protearum* and *Pestalotiopsis* spp., which were the predominant species in the roots, stems, and hypocotyl tissues. Some predominant endophytes exhibited high tissue specificity, e.g., *Phomopsis longicolla*, *Lasiodiplodia pseudotheobromae*, and *Diaporthe eucalyptorum*. These were determined to be the predominant species in the stems, but they were not detected in any other tissue types (Figs 1 and 2A).

**Table 3:** Dominant endophytes and relative species frequency (RF%) in different tissues of each host species.

Host plant	Tissues	Segments examined	Total isolates	Endophytes species	Dominant endophyte	RF%
*R*. *stylosa*	Root	75	25	9	*Neofusicoccum mangiferae*	28.00
					*Neopestalotiopsis protearum*	20.00
					*Pestalotiopsis sp*.	16.00
					*Botryosphaeria dothidea*	8.00
	Stem	75	54	16	*Neopestalotiopsis protearum*	22.22
					*Phomopsis longicolla*	14.81
					*Lasiodiplodia pseudotheobromae*	12.96
					*Pestalotiopsis sp*.	11.11
					*Diaporthe eucalyptorum*	7.41
					*Diaporthe perseae*	7.41
	Leaf	75	3	3	*-*	*-*
	Hypocotyl	75	48	11	*Neopestalotiopsis protearum*	50.00
					*Diaporthe perseae*	12.50
					*Pestalotiopsis sp*.	12.50
	Flower	75	5	5	*-*	*-*
*R*. *mucronata*	Root	75	26	9	*Lasiodiplodia theobromae*	34.62
					*Colletotrichum gloeosporioides*	15.38
					*Diaporthe pascoei*	15.38
					*Diaporthe sp*.	15.38
	Stem	75	44	14	*Neopestalotiopsis protearum*	15.91
					*Pestalotiopsis sp*.	13.64
					*Diaporthe pascoei*	11.36
					*Lasiodiplodia theobromae*	11.36
					*Neofusicoccum mangiferae*	11.36
					*Neofusicoccum parvum*	9.09
	Leaf	75	4	1	*-*	*-*
	Flower	75	16	9	*Diaporthe sp*.	25.00
					*Neofusicoccum mangiferae*	25.00

### Phylogenetic diversity of culturable fungi derived from *R*. *mucronata*

**Fig 3:**
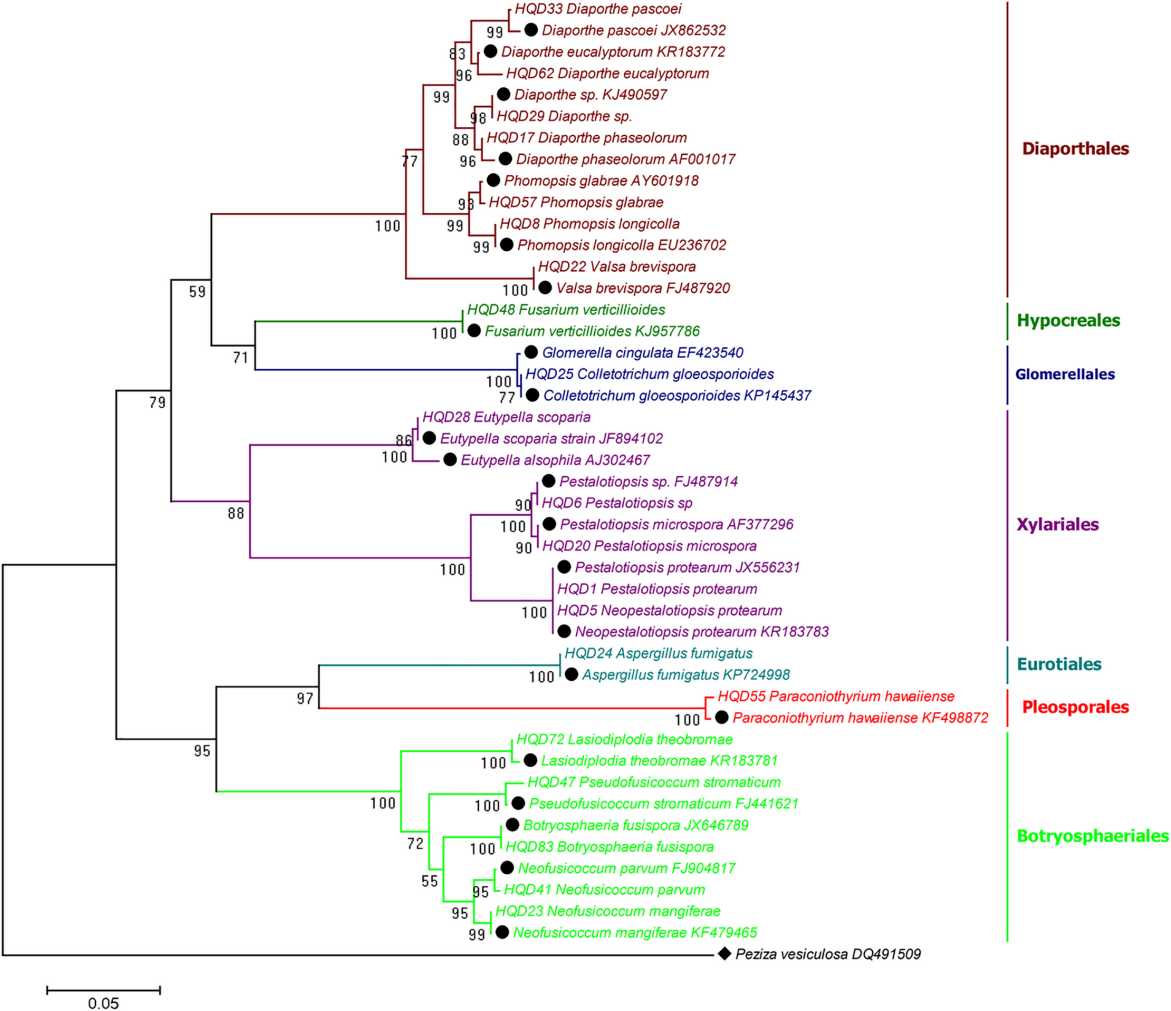
Neighbor-joining phylogenetic tree of 21 representative isolates from *R*. *mucronata* based on ITS gene sequences. The values at each node represent the bootstrap values from 1,000 replicates, and the scale bar represents 0.05 substitutions per nucleotide. *Peziza vesiculosa* DQ491509 served as an outgroup.

With regards to *R*. *mucronata*, 21 isolates representing 90 endophytic fungi were sequenced using the same approach as *R*. *stylosa* (S2 Fig). We were able to further categorize these isolates into 13 genera, namely, *Aspergillus*, *Botryosphaeria*, *Colletotrichum*, *Diaporthe*, *Eutypella*, *Fusarium*, *Lasiodiplodia*, *Neofusicoccum*, *Paracamarosporium*, *Pestalotiopsis*, *Phomopsis*, *Pseudofusicoccum*, and *Valsa*, which belong to seven orders, Botryosphaeriales, Diaporthales, Eurotiales, Glomerellales, Hypocreales, and Pleosporales, all of which are in phylum Ascomycota. *Diaporthe* and *Pestalotiopsis* were the predominant isolated fungal genera (RF = 20.94%), followed by *Neofusicoccum* (RF = 20.93%). Although *Lasiodiplodia* accounted for 17.44% of the total isolates, only one species from this genus was present. Of the 21 identified fungal isolates, *Lasiodiplodia theobromae*, *Neofusicoccum mangiferae*, *Diaporthe pascoei*, *Neopestalotiopsis protearum*, *Pestalotiopsis* spp., *Diaporthe* spp., and *Neofusicoccum parvum* occurred most frequently, accounting for 17.4%, 15.1%, 10.5%, 8.1%, 8.1%, 7.0%, and 5.8% of the total isolates, respectively (Table 2, Figs 2B and 3). The colonization frequencies of the fungal endophytes from different tissues showed significant differences, in the following decreasing order: stems (CF = 44.0%) > roots (26.7%) > flowers (16.0%) > leaves (5.3%) (Table 3). The stems thus exhibited the highest endophytic fungal colonization frequency, as observed in *R*. *stylosa*.

### Comparison of culturable fungal diversity in two mangrove species

We selected the native mangrove species *R*. *stylosa* and an exotic mangrove species introduced from Australia to the South China Sea, *R*. *mucronata* and investigated their endophytic community diversity. The results indicated that the 46 independent representatives out of the 220 isolates from *R*. *stylosa* and *R*. *mucronata* that were selected for sequencing represented 17 genera belonging to eight taxonomic orders of Ascomycota. *Pestalotiopsis* sp. (34.54%), *Diaporthe* (18.62%), and *Neofusicoccum* (14.54%) were found to be the predominant culturable genera. The most frequent colonizers of *R*. *stylosa* and *R*. *mucronata* were *Neopestalotiopsis protearum* and *Lasiodiplodia theobromae*, respectively. These representative isolates and their best matches in the NCBI database are summarized in Table 2. Most of the isolates matched their closest relatives with 98%–100% homology, with the exception of HHL052 (96%), HHL053 (97%), HHL081 (97%), and HQD62 (97%). Four orders, Botryosphaeriales, Diaporthales, Hypocreales, and Xylariales, seven genera (*Botryosphaeria*, *Diaporthe*, *Fusarium*, *Neofusicoccum*, *Pestalotiopsis*, *Phomopsis*, and *Valsa*) and eight species (*Diaporthe eucalyptorum*, *Lasiodiplodia theobromae*, *Neofusicoccum mangiferae*, *Neofusicoccum parvum*, *Pestalotiopsis microspora*, *Phomopsis glabrae*, *Phomopsis longicolla*, and *Valsa brevispora*) were commonly isolated from both mangroves, with the majority of the fungi belonging to Diaporthales and Xylariales (Table 2, Figs 1 and 2).

To characterize the endophytic community, isolates from the various tissues were compared using the Shannon-Wiener and Simpson’s diversity indices (Table 4). The Shannon-Wiener diversity index (*H*′) indicated that *R*. *mucronata* (2.63) exhibited higher fungal species diversity than *R*. *stylosa* (2.52), and similarly, Simpson’s diversity index (1−*D*) suggested higher fungal endophyte diversity in *R*. *mucronata* (0.91) than in *R*. *stylosa* (0.86). The Shannon and Simpson diversity indices were highest in the stems than the other tissues in both mangrove species (*H*′ = 1.32 and 1.54; 1−*D* = 0.88 and 0.90), suggesting that these endophytic fungi have a preference for stem colonization. The Shannon evenness (*E*) index of *R*. *mucronata* (0.85) was similar to that of *R*. *stylosa* (0.78), indicating uniform species composition across both hosts. The values obtained in these analyses indicate that the endophytic fungal diversity in *R*. *mucronata* and *R*. *stylos*is is relatively high. The Sorensen’s similarity indices (*QS*) between the endophytes of both mangroves and various tissues ranged from 0.12% to 0.47% (Table 5), indicating significant variation in the endophytic fungal biodiversity from the different tissues of both plants. We used three different estimators based on abundance data. The observed number of operational taxonomic units (OTUs) was still lower than predicted in estimates using the nonparametric ACE, Chao1, and Chao2 indices, with values of 36.12, 33.04, and 36.29 for *R*. *stylosa*, and 25.59, 24.56, and 24.36 for *R*. *mucronata*, respectively, at 97% similarity.

**Table 4:** Colonization frequency (%CF) and diversity indices of endophytic fungi isolated from different tissues of *R*. *stylosa* and *R*. *mucronate*.

Indices	*R*. *stylosa*						*R*. *mucronata*				
	Root	Stem	Leaf	Hypocotyl	Flower	Total	Root	Stem	Leaf	Flower	Total
Colonization frequency(%)	25.33	53.33	4.00	49.33	6.67	27.73	26.67	44.00	5.33	16.00	23.00
Shannon-Wiener	1.96	2.39	1.10	1.69	1.61	2.52	1.86	2.42	-	2.08	2.63
Simpson’s diversity	0.83	0.88	0.67	0.71	0.80	0.86	0.80	0.90	-	0.84	0.91
Shannon eveness (*E*)	0.61	0.74	0.34	0.52	0.50	0.78	0.61	0.79	-	0.68	0.86
Camargo’s index (1/*S*)	0.11	0.06	0.33	0.09	0.20	0.04	0.11	0.07	1.00	0.11	0.05
ACE						36.12					25.59
Chao-1						33.04					24.56
Chao-2						36.29					24.36

**Table 5:** Sorenson’s similarity indices for endophytic fungi from different tissues of *R*. *stylosa* and *R*. *mucronate*.

		*R*. *stylosa*					
		Root	Stem	Leaf	Hypocotyl	Flower	Plant
***R*. *mucronata***	Root	0.00	0.32	0.00	0.20	0.00	0.24
	Stem	0.35	0.47	0.12	0.32	0.21	0.46
	Leaf	0.20	0.00	0.00	0.17	0.33	0.08
	Flower	0.22	0.24	0.00	0.20	0.14	0.29
	Plant	0.27	0.43	0.08	0.25	0.15	0.43

### Screening for antioxidant activity in endophytic fungal extracts

Another aim of the present study was to identify the antioxidant potential of endophytic fungi from the two mangrove species. A total of 46 representative endophytic fungal extracts from *R*. *stylosa* and *R*. *mucronata* were tested for antioxidant capacity, including the ability to scavenge DPPH and ABTS radicals using a microtiter plate assay. Of the four media used for fermentation, Czapek’s agar and rice medium were determined to be more suitable for antioxidant production in fungal isolates than PDA and the grain medium for both host plants. Most of the fungal strains (39 isolates, 84.8%) exhibited antioxidant capacity to some extent (%RSA > 50%) and a concentration of 10 mg/mL was selected based on an antioxidant activity assay using different concentrations (1 mg/mL to 10 mg/mL). The DPPH and ABTS radical scavenging activities of the crude extracts showed a dose-dependent relationship. Their IC_50_ values were obtained by interpolation from a linear regression analysis, which indicated that each isolate had high levels activity in one or more of the different culture media. High rates of antioxidant activity were detected from the fungal extracts belonging to Diaporthales and Xylariales. Four culture media of *Diaporthe eucalyptorum* (HHL53 and HHQ62) and *Cytospora rhizophorae* (HHL55) all exhibited moderate scavenging ability towards DPPH or ABTS radicals. Czapek’s agar culture of the endophytic strains *Cytospora rhizophorae* (HHL55) and *Seiridium ceratosporum* (HHL38) isolated from *R*. *stylosa* showed the most potent radical-scavenging activity, with IC_50_ values of 0.33±0.02 mg/mL and 0.37±0.02 mg/mL, respectively (Table 6, S3 Fig). The radical scavenging activities of these fungal extracts were comparable to ascorbic acid (Vc, IC_50_ of 0.10±0.01 and 0.09±0.00 mg/mL), suggesting their potency. Further research also provided the first evidence that HQD-6 could produce flufuran, which would then act as a scavenger of DPPH and ABTS radicals, with IC_50_ values of 34.85±1.56 and 9.75±0.58 μg/mL, respectively (NMR data elsewhere), indicating that it is more potent as an antioxidant than the positive control.

**Table 6:** IC_50_ values from antioxidant activity assays from isolates of *R*. *stylosa* and *R*. *mucronate*.

Isolate ID	Fungal isolate	IC_50_(mg/ml)							
		DPPH				ABTS			
		PDA	CZA	RM	GM	PDA	CZA	RM	GM
HHL104	*Cladosporium cladosporioides*	-	-	-	-	-	1.47±0.02	-	9.88±1.46
HHL55	*Cytospora rhizophorae*	-	0.33±0.02	0.58±0.01	0.65±0.05	5.46±0.63	0.50±0.01	1.59±0.06	1.17±0.04
HHL59	*Diaporthe ceratozamiae*	-	-	-	-	1.80±0.05	-	-	-
HHL53	*Diaporthe eucalyptorum*	-	-	-	-	1.03±0.02	4.18±0.20	0.77±0.03	-
HHL61	*Diaporthe perseae*	-	1.21±0.09	-	-	-	1.75±0.06	-	-
HHL7	*Diaporthe sp*.	2.19±0.07	-	-	9.97±0.78	1.67±0.39	2.92±0.14	-	2.73±0.13
HHL48	*Fusarium solani*	-	-	-	-	-	-	-	4.11±0.55
HHL96	*Guignardia mangiferae*	-	-	5.49±0.39	-	-	-	1.80±0.10	-
HHL31	*Lasiodiplodia pseudotheobromae*	-	-	-	-	-	-	-	10.17±0.23
HHL94	*Lasiodiplodia theobromae*	-	-	14.36±0.68	3.24±0.11	-	-	-	1.48±0.13
HHL129	*Neofusicoccum mangiferae*	-	-	-	-	-	-	-	6.78±0.39
HHL75	*Neofusicoccum parvum*	-	3.62±0.38	-	-	-	3.65±0.46	-	-
HHL46	*Neopestalotiopsis protearum*	1.80±0.05	3.13±0.30	-	-	1.24±0.14	1.37±0.07	-	5.13±0.18
HHL82	*Pestalotiopsis microspora*	-	-	2.33±0.17	-	2.99±0.29	1.93±0.06	1.95±0.15	-
HHL79	*Pestalotiopsis photiniae*	-	-	-	-	-	-	-	9.01±0.57
HHL56	*Pestalotiopsis theae*	3.82±0.46	-	-	-	1.12±0.03	7.87±0.59	-	-
HHL52	*Phomopsis glabrae*	-	-	0.86±0.43	-	-	-	1.29±0.09	-
HHL50	*Phomopsis longicolla*	4.60±0.51	1.40±0.14	-	-	1.98±0.08	1.03±0.04	-	5.49±0.72
HHL20	*Phomopsis sp*.	-	-	11.94±1.30	-	-	-	5.20±0.81	-
HHL38	*Seiridium ceratosporum*	-	3.76±0.07	-	-	-	0.37±0.02	-	-
HHL81	*Valsa brevispora*	3.05±0.12	-	0.43±0.06	6.27±1.66	1.46±0.10	-	0.76±0.23	2.85±0.21
HQD24	*Aspergillus fumigatus*	-	-	-	-	-	1.55±0.03	-	-
HQD83	*Botryosphaeria fusispora*	-	2.47±0.43	-	-	-	3.10±0.20	-	-
HQD25	*Colletotrichum gloeosporioides*	-	4.56±0.42	-	-	-	3.52±0.63	-	-
HQD62	*Diaporthe eucalyptorum*	-	-	-	-	3.00±0.15	1.03±0.02	0.77±0.03	1.17±0.04
HQD33	*Diaporthe pascoei*	-	4.06±0.29	1.79±0.17	-	13.56±3.55	5.47±0.27	2.32±0.20	-
HQD17	*Diaporthe phaseolorum*	5.27±1.80	5.03±0.82	4.64±0.35	7.57±1.55	4.8±0.99	1.67±0.17	2.40±0.11	-
HQD29	*Diaporthe sp*.	9.64±0.85	9.97±1.17	0.95±0.03	-	4.89±0.16	3.88±0.63	1.32±0.04	2.62±0.11
HQD28	*Eutypella scoparia*	-	-	-	-	-	8.74±0.34	-	-
HQD72	*Lasiodiplodia theobromae*	-	-	4.12±0.47	1.45±0.04	-	-	-	1.48±0.13
HQD23	*Neofusicoccum mangiferae*	-	-	-	-	-	-	-	6.78±0.39
HQD41	*Neofusicoccum parvum*	-	3.7±0.33	-	-	-	3.65±0.46	-	-
HQD5	*Neopestalotiopsis protearum*	-	-	-	-	1.24±0.14	1.37±0.07	-	5.13±0.18
HQD55	*Paraconiothyrium hawaiiense*	-	-	-	-	-	-	2.95±0.12	-
HQD20	*Pestalotiopsis microspora*	-	-	2.33±0.17	-	2.99±0.29	1.93±0.06	1.95±0.15	-
HQD6	*Pestalotiopsis sp*.	-	-	0.65±0.19	1.06±0.01	10.09±1.1	-	0.75±0.11	1.13±0.05
HQD57	*Phomopsis glabrae*	-	-	1.23±005	-	-	-	1.29±0.09	-
HQD8	*Phomopsis longicolla*	-	-	-	-	1.98±0.08	1.03±0.04	-	5.49±0.72
HQD47	*Pseudofusicoccum stromaticum*	-	5.20±0.84	-	-	-	2.16±0.06	-	1.68±0.03
HQD22	*Valsa brevispora*	4.31±0.65	-	1.82±0.03	9.68±2.44	1.46±0.1	-	0.76±0.23	2.85±0.21
Vc		0.10±0.01				0.09±0.00			

## Discussion

Mangroves are coastal biotopes that develop within the intertidal zone. They are tolerant of many types of extreme environmental stress. They harbor diverse fungal communities, which makes them potential sources of bioactive natural products. The present study showed that the endophytic fungal community of various tissues from *R*. *stylosa* and *R*. *mucronata* to be highly diverse, and a total of 46 independent representative species were isolated. The leaf endophyte composition of five *Sonneratia* mangrove species in the South China Sea [21], endophytic fungi of *R*. *mucronata* of Thailand [34] and two *Rhizophora* species (*R*. *apiculata* and *R*. *mucronata*) [35] growing in the New Caledonia mangrove forest in France all differed greatly from the present study. Phylogenetic analysis based on the ITS region identified 17 genera belonging to eight taxonomic orders within Ascomycota, with *Pestalotiopsis* and *Diaporthe* as the most prevalent endophytes. This is consistent with the findings of some previous studies [36, 37].

Previous phylogenetic studies have suggested that host specificity is relatively common at higher levels of the host taxon [38, 39]. In our study, a significant proportion of the predominant isolates, including eight species belonging to seven genera (*Diaporthe eucalyptorum*, *Lasiodiplodia theobromae*, *Neofusicoccum mangiferae*, *Neofusicoccum parvum*, *Pestalotiopsis microspora*, *Phomopsis glabrae*, *Phomopsis longicolla*, and *Valsa brevispora*) were found to be common in both *R*. *stylosa* and *R*. *mucronata*, which supports the conclusion that endophytic fungi may be more common at the host family level than at the host species level. The remaining species demonstrated host specificity, but not preference for any particular host species. Some of the isolates that have been previously reported as endophytes, e.g., *Seiridium ceratosporum*, *Cytospora rhizophorae*, *Pseudofusicoccum stromaticum*, and *Paraconiothyrium hawaiiense*, were observed in mangroves for the first time in this work.

The diversity indices served as evidence of the diversity within the composition of the endophytic fungal community in *R*. *stylosa* and *R*. *mucronata*, and the distribution of these endophytic fungi was tissue-specific. The relative frequency of colonization of endophytic fungi in different tissues was in the order of stems > hypocotyls > roots > flowers > leaves, which is consistent with the findings of previous reports [21]. Several endophytes, including *Colletotrichum*, *Eutypella*, and *Neofusicoccum*, were specifically distributed in the roots but did not colonize the stems or other tissues. The tissue-specific differences in microbial community suggest preference for distinct microenvironments. Analysis of nonparametric ACE, Chao1, and Chao2 indices indicated statistically sufficient sampling for the species composition analysis. Several factors may affect species composition, including seasonal changes, host age, and collection site. Culture-independent fungi were not considered in the present study [40] Culture-free methods including metagenomics analysis, sampling total DNA directly from the environment, and the heterologous expression of a biosynthetic gene in yeast or bacteria may be utilized as a complementary approach to conventional cultivation for the examination of fungal communities [41].

There has been little research into the antioxidants derived from mangrove endophytic fungi. In the present study, the antioxidant activities of various endophytic fungal extracts from *Rhizophora* plants were investigated. *R*. *apiculata* may be an excellent source of antioxidants. Various *in vitro* assays and *in vivo* studies have shown that the crude extracts of *R*. *apiculata* exhibit strong antioxidant activity, thereby imparting a protective effect on sodium nitrite-induced oxidative stress in the brains of rats [42, 43]. *R*. *mucronata* has been shown to have highly effective scavenging towards DPPH, hydroxyl, nitric oxide radicals, and hydrogen peroxide radicals [44, 45]. To efficiently utilize the mangrove fungal sources available from a single fungal strain, we attempted to explore the antioxidants of isolated fungi by changing the cultivation media. Four culture media, including PDA, CZA, RM, and GM, were tested using improved DPPH and ABTS assays. Among the four media used for fermentation, CZA and RM were demonstrated to have the highest suitability for antioxidant production. The Czapek’s agar culture of the endophytic strains *Cytospora rhizophorae* (HHL55) and *Seiridium ceratosporum* (HHL38) isolated from *R*. *stylosa* were found to be the most potent scavengers of DPPH and ABTS radicals. Their differences might be ascribed the use of different culture media to activate biosynthetic gene clusters to produce the improved cryptic antioxidant [46]. A large proportion (84.8%) of fungal isolates possessed strong antioxidant capacities (%RSA > 50%), indicating that these plants and their endophytes might serve as sources of natural antioxidants.

Previous reports have described the antioxidant potential of some endophytic fungal metabolites including isobenzofurans pestacin and isopestacin isolated from *Pestalotiopsis microspore*, which was derived from *Terminalia morobensis*, and the strong free radical-scavenging agent graphislaetone A, which was isolated from *Trachelospermum jasminoides* endophytic *Cephalosporium* sp. IFB-E001 [47–49]. However, few natural antioxidants have been identified from mangrove endophytic fungi. One of the few confirmed antioxidants is xyloketals B, produced by the unidentified fungal strain *Xylaria* sp. No. 2508, which colonizes in *Avicennia marina*. It has been demonstrated to be a natural antioxidant and it could protect OGD-induced and MPP+-induced neurotoxicity in *Caenorhabditis elegans* and PC12 cells through the restoration of total GSH levels and through its antioxidant property [50, 51]. Because of these findings, researchers have subsequently focused on identifying the antioxidants associated with mangrove endophytic fungi. This has led to the isolation of a new compound with antioxidant activities, xyloketal B, isolated from the mangrove fungus *Xylaria* sp., which imparts protection against oxLDL-induced endothelial oxidative injury possibly by inhibiting NADPH oxidase-derived ROS generation, promoting NO production, and restoring Bcl-2 expression, thereby rendering it a promising candidate for antioxidant discovery [14]. Racemic cyclohexenone and cyclopentenone derivatives, (±)-(4R*,5S*,6S*)-3-amino-4,5,6-trihydroxy-2-methoxy-5-methyl-2-cyclohexen-1-one and (±)-(4S*,5S*)-2,4,5-trihydroxy-3-methoxy-4-methoxycarbonyl-5-methyl-2-cyclopenten-1-one isolated from the culture of the mangrove endophytic fungus *Alternaria* sp. R6, exhibited potent ABTS scavenging activities with EC_50_ values of 8.19 ± 0.15 and 16.09 ± 0.01 μM, respectively [52]. Some studies have suggested that plants associated with salt tolerance and endophytes are typically rich in antioxidants. Mangrove endophytic fungus *Aspergillus flavus* were analyzed using various *in vitro* assay systems, such as iron chelating capacity, reducing power, and hydroxyl radicals/hydrogen peroxide/DPPH radical scavenging and inhibition of lipid peroxidation using the β- carotene-linoleate model system, clearly established the antioxidant potency of extracts of mangrove plants and respective endophytic fungi and the possible roles of antioxidants in symbiotic host-endophytic relationships [53, 54].

## Conclusion

In the present study, a wide variety of culturable fungi from at least 17 genera belonging to eight taxonomic orders of *Ascomycota* was isolated from two *Rhizophora* species. Furthermore, these species are potential sustainable alternative resource for natural antioxidants. Of all the 46 fungal endophytes, HHL55 and HHL38 showed the most potent antioxidant properties. Chemical investigation revealed flufuran as a significant DPPH and ABTS radical scavenger isolated from HQD-6. A detailed investigation of the characterization of antioxidant compounds present in mangrove endophytes must be conducted, other specific metabolites which could explain the significant antioxidant activities of the abovementioned fungal strain must be identified, and new types of biological tests should be established.

## Supporting information

S1 Fig Morphological photos of 25 cultivable fungi associated with mangroves *R. stylosa*. (RAR)

S2 Fig Morphological photos of 21 cultivable fungi associated with mangroves *R. mucronata*. (RAR)

S3 Fig DPPH, and ABTS radical-scavenging capacity of fungal extracts from *R*. *stylosa* and *R*. *mucronata* cultured on: (A) potato dextrose agar (PDA); (B) Czapek’s agar; (C) rice medium; (D) grain medium.
(RAR)
